# Infectious Disease and Grouping Patterns in Mule Deer

**DOI:** 10.1371/journal.pone.0150830

**Published:** 2016-03-23

**Authors:** María Fernanda Mejía Salazar, Cheryl Waldner, Joseph Stookey, Trent K. Bollinger

**Affiliations:** 1 Department of Veterinary Pathology, University of Saskatchewan, Saskatoon, SK, Canada; 2 Department of Large Animal Clinical Sciences, University of Saskatchewan, Saskatoon, SK, Canada; 3 Canadian Wildlife Health Cooperative (CWHC), Saskatoon, SK, Canada; University of Pretoria, SOUTH AFRICA

## Abstract

Infectious disease dynamics are determined, to a great extent, by the social structure of the host. We evaluated sociality, or the tendency to form groups, in Rocky Mountain mule deer (*Odocoileus hemionus hemionus*) from a chronic wasting disease (CWD) endemic area in Saskatchewan, Canada, to better understand factors that may affect disease transmission. Using group size data collected on 365 radio-collared mule deer (2008–2013), we built a generalized linear mixed model (GLMM) to evaluate whether factors such as CWD status, season, habitat and time of day, predicted group occurrence. Then, we built another GLMM to determine factors associated with group size. Finally, we used 3 measures of group size (typical, mean and median group sizes) to quantify levels of sociality. We found that mule deer showing clinical signs of CWD were less likely to be reported in groups than clinically healthy deer after accounting for time of day, habitat, and month of observation. Mule deer groups were much more likely to occur in February and March than in July. Mixed-sex groups in early gestation were larger than any other group type in any season. Groups were largest and most likely to occur at dawn and dusk, and in open habitats, such as cropland. We discuss the implication of these results with respect to sociobiology and CWD transmission dynamics.

## Introduction

The rate and pattern of information and pathogen spread within a population depends on its social structure [1, 2]. Grouping patterns are useful to describe social structure [3] and have implications for disease spread [4]. Here, we investigate whether chronic wasting disease (CWD) infection influences grouping patterns in a free-ranging mule deer (*Odocoileus hemionus hemionus*) population. We determine factors that predict group size and occurrence, and describe group size distribution to provide empirically derived parameters for CWD transmission models.

CWD is a fatal, neurodegenerative, contagious prion disease that affects mule deer, white-tailed deer (*Odocoileus virginianus*), elk (*Cervus canadensis*) and moose (*Alces alces*) in North America. CWD has a long incubation period (about 17 months) during which infectious prions are shed in saliva, urine and feces [5]. Transmission occurs by direct animal to animal contact or by contact with prion contaminated environments [6]. Although rates of shedding are unknown, they are assumed to be greatest during the later clinical phase of disease, which lasts from few weeks to around 4 months [7]. Changes in social behavior of the host (e.g. interactions with other deer) at the early stages of clinical disease are very subtle and have only been mentioned by animal handlers familiar with captive individuals [8]. These, and other behavioral changes such as stereotypic movements (repetitive head tossing and exaggerated gait movements), diminished alertness, hyperphagia and polydipsia [5], can probably be attributed to lesions on certain regions of the brain caused by the accumulation of disease-associated prion protein [8].

CWD is a serious concern for wildlife management agencies for various reasons. It has caused considerable economic, ecological, and cultural impact [9], and poses a potential risk for human and livestock health if it eventually crosses the species barrier [10]. Moreover, control efforts to date have not been successful, resulting in continued geographic spread. As disease prevalence increases, herds of cervids infected with CWD could be extirpated [11]. A better understanding of transmission dynamics is needed to develop well-informed epidemiologic models and effective control strategies. However, there are several sources of complexity when modeling transmission dynamics of CWD. These include seasonal movement patterns of the host, habitat selection, contamination and persistence of prions in the environment, and transmission through individual contacts governed by cervid sociality [12, 13].

Since social animals transfer both information and pathogens by means of relationships, the probability of becoming infected increases in larger groups, and risk of infection could therefore be seen as a cost of sociality [14]. However, empirical evidence for this feature is mixed [4, 15], and the relationships between epidemiological and ecological processes is intricate [16]. For example, group size is positively correlated with increased prevalence (percentage of infected hosts) and intensity (number of parasites in each infected host) of contact-transmitted parasites in a variety of species [17]. Yet, infection rates may [18] or may not be [19] explained by measures of social connectivity such as network density and clustering coefficients. Equally important is that social behaviors that restrict the spread of pathogens have evolved in animal populations and have consequences on pathogen transmission dynamics [20]. These behavioral strategies can be noted in both infected and healthy individuals [21–23].

Host group size is an important factor in various measures of disease transmission. For instance, the probability of a pandemic occurring, the average number of groups infected by the initial group, and the proportion of the population infected over the course of an epidemic, depend on group size, among other factors such as rate of mixing among groups, and length of infectious period [24]. The long incubation period of CWD [7] adds complexity to epidemiological processes, as during the majority of this time deer are capable of directly transmitting the disease and shed prions into the environment [25], where prions remain bioavailable for at least 2.5 years [6].

As noted by Potapov et al in 2013 [26] and Oraby et al in 2014 [27], validity of CWD transmission model outcomes can depend on the level of detail of the data on deer social behavior and dynamics of prions in the environment that are used for parameter estimation. Failure to understand factors affecting social behavior limits the applicability and introduces error into disease models designed to inform and guide control strategies. Many important aspects of mule deer social behavior have been described [28–31]. Mule deer are variably gregarious, showing a great frequency of solitary individuals (up to 64%) and small groups (60.8% to 78% of groups with ≤5 deer) [28, 29]. Group size and distribution of mule deer vary seasonally and with habitat conditions [29, 31]. Even though group size increases with habitat openness and with distance from closed habitats, it is not affected by habitat patch size, preferred forage availability, closeness to water sources, or terrain steepness and ruggedness [29]. With respect to group stability, both cohesiveness of groups with mule deer fawns, and frequency of associations between fawns increase over winter [31]. It is not yet known how CWD infection affects mule deer grouping patterns after accounting for factors such as sex, age, season, time of day, and habitat. This is probably why current CWD models, no matter how complicated or simple they are, either rely on simplified dynamics of mule deer sociality [26, 27] or do not attempt to account for social structure [32–34].

We hypothesized that CWD infection affects mule deer social behavior, thereby clinically sick individuals would be more likely to occur in solitude than clinically healthy individuals. We also anticipated that group size would vary across seasons and habitats, so that largest groups would happen in winter and in open habitats, as previously reported in other geographical locations [28, 29, 31]. To answer these questions we analyzed grouping patterns of a mule deer population in a CWD endemic area. Our objectives were to 1) examine whether factors, such as season, habitat, and presentation of CWD clinical signs, were associated with deer being grouped or alone, 2) examine if the same factors were associated with group size, and 3) quantify levels of sociality by calculating 3 measures of group size (typical, mean and median group sizes).

## Materials and Methods

### Study population

The study was conducted at Antelope Creek (50.66°N, 108.27°W) in south Saskatchewan, Canada, between 2007 and 2013. This area includes a 248 km^2^ rural area within the mixed grassland ecoregion. The most common habitat is crop (46.6%), followed by grassland (35.6%), shrub (7.8%), mixed grassland and shrub (7.6%), woodland (2%) and open water (0.3%). The study population was composed of free ranging Rocky Mountain mule deer, with 67% of adults being non-migratory [35]. Their predominant predators are coyotes (*Canis latrans*) and humans (*Homo sapiens sapiens*), as black bears (*Ursus americanus*) and gray wolves (*Canis lupus*) have been extirpated from the area. First recognized in Antelope Creek in 1996, CWD prevalence in adult mule deer increased from 0.98% (20/2046) in 2004 to 6.5% (16/246) in 2009 [36].

### Data collection

This study was approved by the University of Saskatchewan’s Animal Research Ethics Board (Permit number 20050135), and adhered to the Canadian Council on Animal Care guidelines for humane animal use. Permits to conduct research within private land of the study area were obtained verbally from land owners. Permit to conduct research within the Cabri Regional Park (GPS: 50.66824–108.26791) was obtained from The Saskatchewan Regional Parks Association.

As part of a study of CWD transmission dynamics, we captured and radio-collared mule deer during 2 time periods each year between 2007 and 2012. In June and July we captured fawns, and between January and April we used helicopter and net guns, or less commonly Clover traps [37], to capture juveniles and adults (Table 1). Captured deer were fitted with either a global positioning system (GPS) or a very-high-frequency (VHF) radio-collar (Lotek Wireless, Ontario, Canada, and Advanced Telemetry Systems, Minnesota, USA). Fawns always received an expandable VHF collar.

**Table 1:** Annual numbers of newly captured (and recaptured) mule deer in Antelope Creek by age and sex class.

	Year of capture						
Age and sex class	2007	2008	2009	2010	2011	2012	Total
**Adult**	32	4	47 (13)	43 (35)	44 (42)	(61)	170 (151)
Female	12	1	25 (6)	15 (25)	7 (37)	(30)	60 (98)
Male	20	3	22 (7)	28 (10)	37 (5)	(31)	110 (53)
**Juvenile**	20	3	11	20 (10)	22 (10)	(7)	76 (27)
Female	10	0	5	10 (5)	11 (4)	(3)	36 (12)
Male	10	3	6	10 (5)	11 (6)	(4)	40 (15)
**Fawn**	0	1	38	41	39	0	119
Female	0	0	20	17	22	0	59
Male	0	0	18	24	17	0	60
**Total**	52	8	96 (13)	104 (45)	105 (52)	(68)	365 (178)

We classified captured mule deer based on age (adult, juvenile, or fawn), sex (male or female), and CWD diagnosis (positive or negative) (explained in detail in the following section). During each season, of each year, individuals were either tracked once or twice a month. Those tracked twice a month included: all CWD positive individuals, all CWD negative individuals of each sex and age class with less than 10 deer, and from the list of CWD negative deer remaining (adults and juveniles only), we did a stratified by sex and age class random selection to obtain 10 individuals from each stratum (i.e. 10 adult males, 10 adult females, 10 juvenile males, and 10 juvenile females). The remaining individuals were tracked at least once a month. Fawns were tracked with their mothers. To avoid double tracking of the same collared deer, observers targeted different deer within a day (hereafter focal deer). We tracked deer every day from December 2008 to July 2009, then on weekdays until March 2012, and during 3 days every 2 weeks until December 2012. During these tracking periods we recorded for each group encountered: date, time, habitat, number of individuals in the group, sex and age class of every individual in the group, location using a hand-held GPS, and the distance from observer. We defined a mule deer group as a spatially cohesive and behaviorally coordinated aggregation of deer, in which every deer was within 10 body lengths of at least one other [29]. To consider all social units relevant to the study of social organization, we also defined a solitary deer as a group of 1 [38, 39]. When possible, we also recorded groups in which no radio-collared deer was present. We observed deer from an average distance of 257±172 m (± SD). All observers received training on monitoring of mule deer groups and used binoculars (10x42) and spotting scopes (15-45x60).

### Factors related to group size and group occurrence

We considered several factors with a potential to influence group size and the occurrence of social groups. These included: time of the day, habitat, year, season, month, group type, observer class, presence of CWD positive deer in the group, presence of deer showing clinical signs of the disease, and age, sex and CWD status of the focal deer. The focal deer in a group with more than one collared deer was the individual targeted to be tracked within that day.

We divided the 24-h day into 5 periods: dawn, before solar noon, after solar noon, dusk, and night. Dawn began with the start of civil twilight and ended 90 min after sunrise; before solar noon started with the end of dawn and ended at solar noon; after solar noon started with the solar noon and ended at the start of dusk; dusk started 90 min before sunset and ended at the end of civil twilight; night was the remaining time between 2 continuous days. The times for the points separating these periods were taken from Swift Current historical data [40].

Habitat types used by mule deer were: grassland, low shrub, tall shrub, woodland, and crop. Grasslands consisted of herbs (small tender plants lacking woody stems, such as grasses and forbs), low shrub habitat consisted of small woody perennial plants with foliage mass close to the ground and several low-branching stems, tall shrub was habitat with shrub that was significantly higher than low shrub, woodland included spaced trees with canopy coverage of 25% to 60%, and crop contained plants grown to be harvested for agricultural use [41]. Any other habitat was assigned the class “other”. Observations of deer in tall shrub habitat were excluded from analysis and descriptive statistics because some deer may have been overlooked in this densely vegetated habitat. Observations of deer fleeing from a location of cover (flushed deer) were also excluded as it is difficult to accurately count and classify all deer in a group under such circumstances.

We divided the year into 5 seasons [12]: early gestation (16 Dec to 31 Mar), late gestation (1 Apr to 15 May), fawning (16 May to 31 Jul), pre-rut (1 Aug to 31 Oct), and rut (1 Nov to 15 Dec). To identify sex and age classes of mule deer within a group we used a key (S1 File). We defined fawns as individuals from 0 to ~9 months old, juveniles from 9 to ~21 months old, and adults from 21 months and older. For both sexes, juveniles were slender and had a narrower face than adults. In contrast to juvenile males, antlers in adult males were branched, had a large base girth and spread wider than the ears.

We classified groups into 7 types according to their composition: solitary male (1 adult male or juvenile male), solitary female (1 adult female or juvenile female), group of males (≥2 adult males or juvenile males; can include fawns of any sex, but not juvenile or adult females), group of females (≥2 adult females or juvenile females; can include fawns of any sex, but not juvenile or adult males), mixed-sex group (at least 1 adult female or juvenile female and 1 adult male or juvenile male; can include any other sex and age class), adult female-fawn/juvenile dyad (2 individuals, 1 is an adult female and the other is a fawn or a juvenile of any sex), and unknown (a group of no other type that contains individuals of unknown sex and/or age class). After collecting data, we classified observers as experienced or inexperienced (observer experience); experienced were those with previous training in deer behavior.

For each animal over 9 months of age, we used tonsil and rectal biopsies from live animals sampled during capture, and brain from dead animals, to test for CWD with an immunohistochemical method [42]. We classified collared deer as positives for CWD from day 1 of the month in which the sample was taken (e.g., if a sample taken on Feb 25 was positive, that deer was considered positive since Feb 1). Deer were classified as not positive until the time they tested positive. Groups were classified as having a known positive when at least 1 collared individual previously diagnosed as CWD positive was present in the group. We also recorded that the group had evidence of clinical signs when any deer in a group was showing signs of clinical disease. Clinical signs considered indicative of CWD included drooping ears and head, laterally wide feet stance, hocks touching, and protruding ribs and sometimes ischial tuberosities [5].

### Group occurrence

We built a generalized linear mixed model (GLMM) using the GLIMMIX procedure [43] in SAS v9.3 to identify factors associated with the occurrence of groups. We considered records of groups (the sampling unit) with at least 1 collared deer and in which the focal deer was not a fawn (n = 2173 groups), as they were never seen alone. The final dataset included 188 radio-collared mule deer. In this mixed-effects model, the outcome was group occurrence, a binary variable defined as whether deer were grouped (≥ 2 deer in the group) or alone. The predictor variables examined were: season, month, CWD diagnosis of focal deer, known positive deer in the group, clinical signs of any deer in the group, year, habitat, time of day, observer experience, and age and sex of the focal deer. We treated the person doing the observation and the focal deer as random effects. The GLMM was based on binomial distribution and logit link function. As season and month were correlated, as well as variables focal deer diagnosis, known positive deer in the group, and clinical signs of any deer in the group, we built 6 competing models starting with each possible combination of the correlated variables (Table 2). To identify the best set of variables for each competing model, we first tested one model with all eligible candidate variables and manually sequentially removed the variable with the highest p-value until variables with P<0.05 remained [44]. We then checked for confounding of predictors that were not significant at P<0.05. If reintroduction of a covariate changed the regression coefficient of other variables of interest by 20% or more, then we considered it as a confounder and kept it in the model regardless of its statistical significance [45]. We assessed all biologically relevant potential two-way interactions between predictors that were significant as fixed effects in the model. The interaction term was retained in the final model if P<0.05 for the type 3 likelihood ratio test. We examined diagnostic residual plots for extreme outliers to assess model fit. After the best set of predictor variables for each competing model was identified, we chose the model with the smallest AICc (2498.07) as best model (Table 2). We calculated the variance partition coefficients (VPC) to describe the variance associated with each random effect ([46], p. 583).

**Table 2:** Competing models to predict group occurrence.

Predictor	Model 1	Model 2	Model 3	Model 4	Model 5	Model 6 (best)
Season	**XX**	**XX**	**XX**			
Month				**XX**	**XX**	**XX**
Focal deer diagnosis	**XX**			**XX**		
Known positive in the group		**XXco**			X	
Clinical signs in the group			**XX**			**XX**
Time of day	**XX**	**XX**	**XX**	**XX**	**XX**	**XX**
Habitat	**XX**	**XX**	**XX**	**XX**	**XX**	**XX**
Focal deer age	**XXco**	**XXco**	**XXco**	X	**XX**	X
Focal deer sex	**XXco**	**XXco**	**XXco**	**XXco**	**XXco**	**XXco**
Observer class	X	X	X	X	X	X
Year	X	X	X	X	X	X
Two-way interactions	**XX**^a^	**XX**^b^	**XX**^c^	X		X
AICc	2544.82	2551.56	2528.99	2507.12	2510.37	2498.07

Predictors marked with X or **XX** were tested in the initial model. Predictors marked with **XX** remained in the final model, some as confounders (co). The AICc corresponds to the best set of predictors for that model. Superscripts indicate significant interaction terms

a = season*habitat, and focal deer diagnosis*focal deer age

b = season*habitat

c = season*habitat, and season*clinical signs.

### Mean group size

We developed a GLMM with the GLIMMIX procedure [43] in SAS v9.3 using a negative binomial distribution and a log link function to assess factors that influence mean group size. All groups (the sampling unit) with at least 1 collared deer (n = 2195 groups) entered the analysis. The final dataset included 197 radio-collared mule deer. In this GLMM, the predictor variables examined were: season, month, CWD diagnosis of focal deer, clinical signs of any deer in the group, year, habitat, group type, time of day, observer experience, and age and sex of the focal deer. We treated the person doing the observation and the focal deer as random effects to account for unmeasured differences among repeated observers and focal deer. We used the same criteria to build and evaluate the models and to check for confounders and interactions as described in the GLMM for group occurrence. As season and month were correlated, as well as focal deer diagnosis and clinical signs of any deer in the group, we built 4 competing models starting with each possible combination of the correlated variables. CWD diagnosis of focal deer and clinical signs were not significant variables (P>0.30). Then, 2 competing models remained (one with season and one with month). We chose the one with the smallest AICc (7778.73 vs 7810.58) as best model. When doing post hoc pairwise testing, we accounted for multiple comparisons using Scheffé’s method. We calculated Lin’s concordance correlation coefficient [47] between predicted and observed group sizes to assess model fit. We calculated a mean-count ratio to denote the variance associated to random effects ([48], p. 697).

### Characterization of group size

Because group size was highly skewed, we used 3 descriptive statistics (typical, mean and median group sizes) to adequately characterize the distribution as recommended by Reiczigel and colleagues [49]. First, we calculated typical group size (TGS) (also called mean crowding [49]) as ∑*Gi*^2^/∑*Gi*, where *Gi* is the size of the *i*th group [50]. This metric is less sensitive to the number of records of solitary animals, and it allowed us to calculate the group size as experienced by a member (any member) within a group. In contrast, we also calculated the mean group size (MGS), which provides the number of animals experienced by an outside observer, such as a predator or a researcher. TGS is generally higher, and never lower, than MGS [3]. We divided the dataset (n = 2656 groups) into 2 parts, one considering groups with at least 1 collared deer (n = 2195 groups), and the other with groups in which none of the members were collared (n = 461 groups). We compared the mean, median, quartiles and frequency distribution of group size in these 2 parts to look at the representation of small groups and determine viability of calculating TGS. As all comparisons lead to the same conclusion that the group size distribution is the same in the 2 parts (data not shown) [51], we proceeded with the calculations using all records (n = 2656 group). Group size distribution of our sample is not normally distributed. Then, as the sample TGS is a biased estimate of the population TGS [52] and sample mean and sample variance are correlated, the most appropriate choice for confidence intervals (CIs) construction is a bias-corrected and accelerated (BCa) bootstrap procedure [53]. We used this method (with 5000 repetitions) to calculate CIs of TGS. We obtained the MGS and its CIs by a BCa bootstrap with 2000 repetitions [53, 54]. And finally, we estimated median group size (median hereafter) and calculated the CIs by a method described by Rózsa et al 2000 [49, 54]. To identify differences in TGS, mean, and median among years, seasons, months, times of the day, habitats, and group types, we used two-sample comparison tests as justified and described in Reiczigel et al 2008 [49] (details in S2 File). All analyses were completed using the freely available software program, QPweb 3.0 [55].

## Results

A total of 4987 groups were observed from 16 February 2008 to 28 November 2013; 2810 records remained after removal of those with deer in tall shrub habitat or that were flushed. We limited the data we used from 16 December 2008 to 15 December 2012 (n = 2656) due to small sample sizes before and after these dates. Mean group size was 3.5 (range = 1 to 39, SD = 3.7), typical group size was 7.3 (95% CI = 6.8 to 8.1), and median group size was 2 (95% CI = 2 to 2). Most groups (83.7%) had 5 or fewer individuals [1 (34.7%), 2 (19.4%), 3 (13.7%), 4 (9.8%), and 5 (6.2%)], and groups with >15 deer were rare (2.1%). Most observed groups were mixed-sex (20.1%), followed by groups of males (19.4%), solitary males (19.2%), groups of females (15.4%), solitary females (15.2%), and adult female-fawn/juvenile dyads (5.9%); we could not assign a sex and age class to every deer in the group in 4.8% of the observations.

### Group occurrence

The best model predicting the occurrence of groups of at least 2 deer included these variables: month, clinical signs, time of day, habitat, and focal deer sex; the latter as a confounder for the effect of habitat. Eight percent of the unexplained variation in group occurrence was explained by the identity of the focal deer, while 6.6% was explained by the person doing the observation. The strength of the associations between these factors and group occurrence is described in Table 3. Healthy looking individuals were more likely to be seen in groups than deer showing clinical signs of CWD. The odds ratios for grouping in any given month (except for June and August) were at least 2 (P≤0.002) times greater than in July. This difference with July was particularly marked in February and March. Mule deer were significantly (P≤0.002) more likely to occur in groups in the most open habitats (crop and grassland) than in the least open habitats (low shrub and woodland).

**Table 3:** The final multivariable model for the occurrence of groups of mule deer describing the strength of association with the presence of deer showing clinical signs, month, time of day, habitat, and sex of focal deer (n = 2173 groups).

Variable		OR	95% CI	P-value
**Clinical signs**				0.0001
yes	no*	2.8	1.66, 4.65	0.0001
**Month**				< .0001
July ^a^	August	1.1	0.76, 1.64	0.59
	September*	2.8	1.77, 4.44	< .0001
	October*	2.0	1.28, 30.50	0.002
	November*	2.9	1.83, 4.55	< .0001
	December*	3.7	2.24, 6.26	< .0001
	January*	8.3	4.58, 15.17	< .0001
	February*	23.3	9.06, 60.14	< .0001
	March*	15.4	7.40, 32.15	< .0001
	April*	7.1	4.09, 12.44	< .0001
	May*	3.3	2.14, 5.17	< .0001
	June	1.3	0.86, 1.85	0.23
**Time of day**				0.01
dawn ^a^	before solar noon	0.8	0.56, 1.03	0.07
	after solar noon	0.9	0.70, 1.28	0.71
	dusk	1.3	0.96, 1.73	0.09
	night	1.7	0.47, 6.50	0.41
**Habitat**				< .0001
grassland ^a^	crop	1.2	0.79, 1.68	0.45
	other	1.1	0.57, 2.31	0.70
woodland ^a^	crop*	4.7	1.80, 12.40	0.002
	grassland*	4.1	1.66, 10.10	0.002
low shrub ^a^	crop*	3.0	1.91, 4.75	< .0001
	grassland*	2.6	1.92, 3.54	< .0001
**Focal deer sex**				0.15
male ^a^	female	1.2	0.93, 1.64	0.15

OR = Odds ratio; CI = Confidence interval

a = reference category.

Variables with significant (P<0.05) odds ratios marked with *.

### Mean group size

The best model predicting group size included the factors season, time of the day, habitat, sex of focal deer, age of focal deer, group type, year, and the interaction terms of season*group type, and season*year. The sex of focal deer was included in the model to adjust for confounding of the effects of habitat and time of day on mean group size. Mean-count ratios were 1.08 for focal deer and 1.1 for observers, indicating a relatively small amount of variability in mean group size among both focal deer and observers. The overall Lin´s concordance correlation coefficient between predicted and observed group sizes was 0.8 (95% CI = 0.7–0.8). Predicted mean group sizes of main effects (except for those included in the interaction terms), with their 95% confidence intervals, are presented in Table 4, and their pairwise comparisons with p-values in Table 5. Groups were larger at dusk than before (P = 0.05) and after solar noon (P = 0.001). Groups were also smaller after solar noon than at dawn (P = 0.01). Group size increased with habitat openness; groups in crop (the most open habitat) were larger than in grassland (P = 0.002) and low shrub (P = 0.0003). Groups with an adult focal deer were larger than those with a juvenile focal deer (P = 0.03).

**Table 4:** Least squares means for predicted mean group size according to time of day, habitat, sex of focal deer, and age of focal deer.

Variable (p-value)^a^	Estimated mean group size	95% CI
**Time of day (P = 0.02)**		
night	2.7	2.0, 3.6
dusk	2.6	2.3, 3.0
dawn	2.5	2.2, 2.9
before solar noon	2.4	2.1, 2.7
after solar noon	2.3	2.0, 2.6
**Habitat (P = 0.001)**		
woodland	2.8	2.1, 3.6
other	2.7	2.3, 3.3
crop	2.7	2.3, 3.1
grassland	2.3	2.0, 2.7
low shrub	2.2	1.9, 2.5
**Focal deer sex (P = 0.11)**		
female	2.6	2.2, 3.0
male	2.4	2.1, 2.8
**Focal deer age (P = 0.02)**		
adult	2.8	2.5, 3.2
juvenile	2.6	2.3, 3.0
fawn	2.2	1.6, 2.9

a = P-values for interaction terms are: season*group type P<0.0001, and season*year P = 0.0004.

**Table 5:** Least squares means differences for predicted mean group size according to time of day, habitat, sex of focal deer, and age of focal deer.

Variable	Relationship	Variable	p-value
**Time of day**			
night	>	dusk	0.78
night	>	dawn	0.62
night	>	before solar noon	0.39
night	>	after solar noon	0.21
dusk	>	dawn	0.39
dusk	>	before solar noon	0.046*
dusk	>	after solar noon	0.001*
dawn	>	before solar noon	0.21
dawn	>	after solar noon	0.01*
before solar noon	>	after solar noon	0.20
**Habitat**			
woodland	>	other	0.93
woodland	>	crop	0.82
woodland	>	grassland	0.17
woodland	>	low shrub	0.06
other	>	crop	0.85
other	>	grassland	0.046*
other	>	low shrub	0.01*
crop	>	grassland	0.002*
crop	>	low shrub	0.0003*
grassland	>	low shrub	0.09
**Focal deer sex**			
female	>	male	0.11
**Focal deer age**			
adult	>	juvenile	0.03*
adult	>	fawn	0.052
juvenile	>	fawn	0.20

Significant (≥0.05) p-values are marked with *.

**Fig 1:**
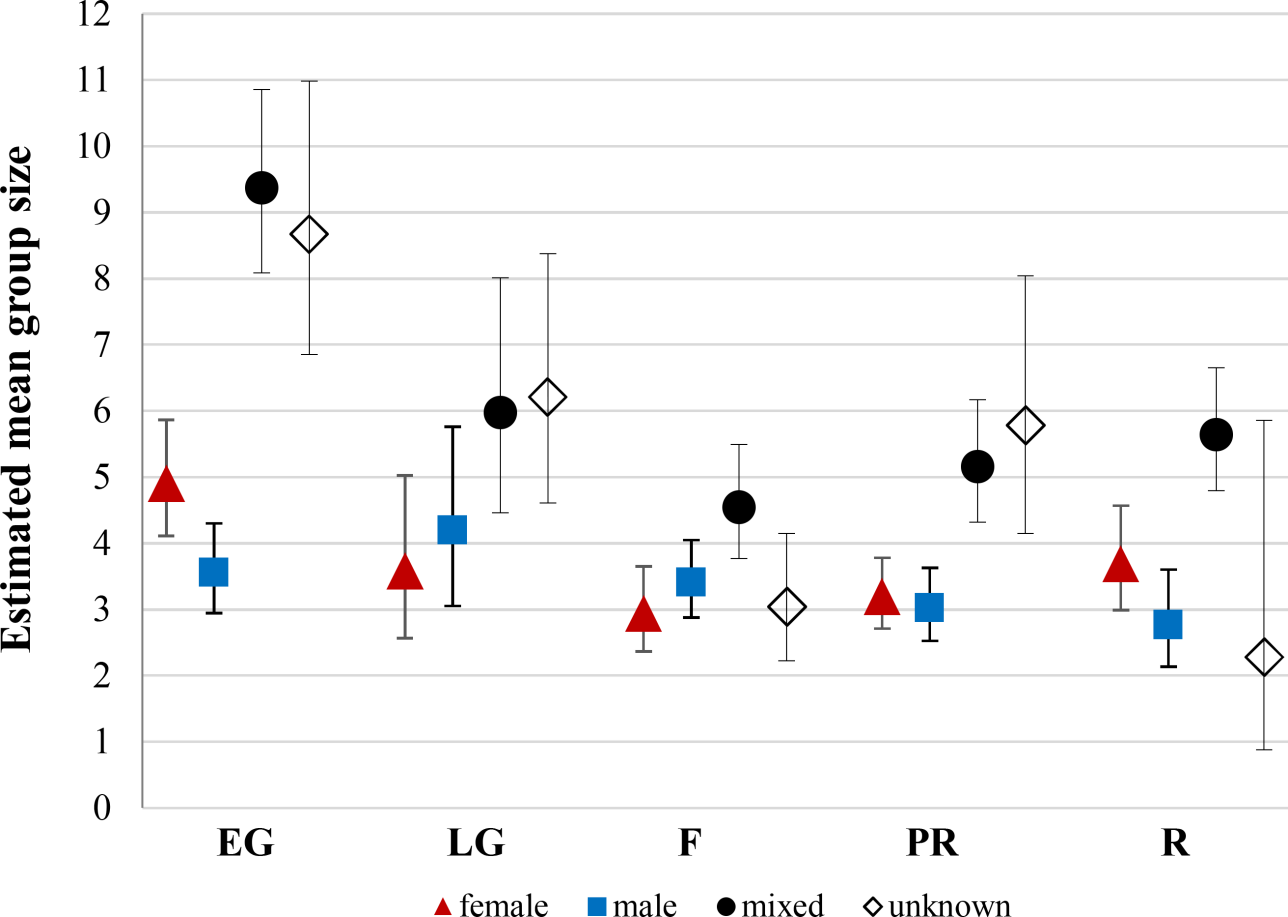
Predicted seasonal variation of mean group size by group type. Bars are 95% confidence intervals. EG = early gestation, LG = late gestation, F = fawning, PR = pre-rut, and R = rut.

Mixed-sex groups were significantly larger than both female and male groups in every season (P<0.001) (Fig 1). The only time in which groups of males were smaller (P = 0.001) than groups of females was in early gestation. The size of female groups was larger in early gestation than in the rest of the seasons (P<0.01), except for late gestation (P = 0.1). Male groups were significantly larger in late gestation than in pre-rut (P = 0.04) and rut (P = 0.03). Mixed-sex groups in early gestation were significantly larger than in the rest of the seasons (P<0.002), and larger in rut than in fawning (P = 0.02). Groups in 2010 during early gestation (P>0.001) and late gestation (P = 0.03) were smaller than in the same seasons in 2011. Groups in rut 2011 were smaller than in rut 2010 (P = 0.005) and rut 2012 (P = 0.03).

### Characterization of group size

**Fig 2:**
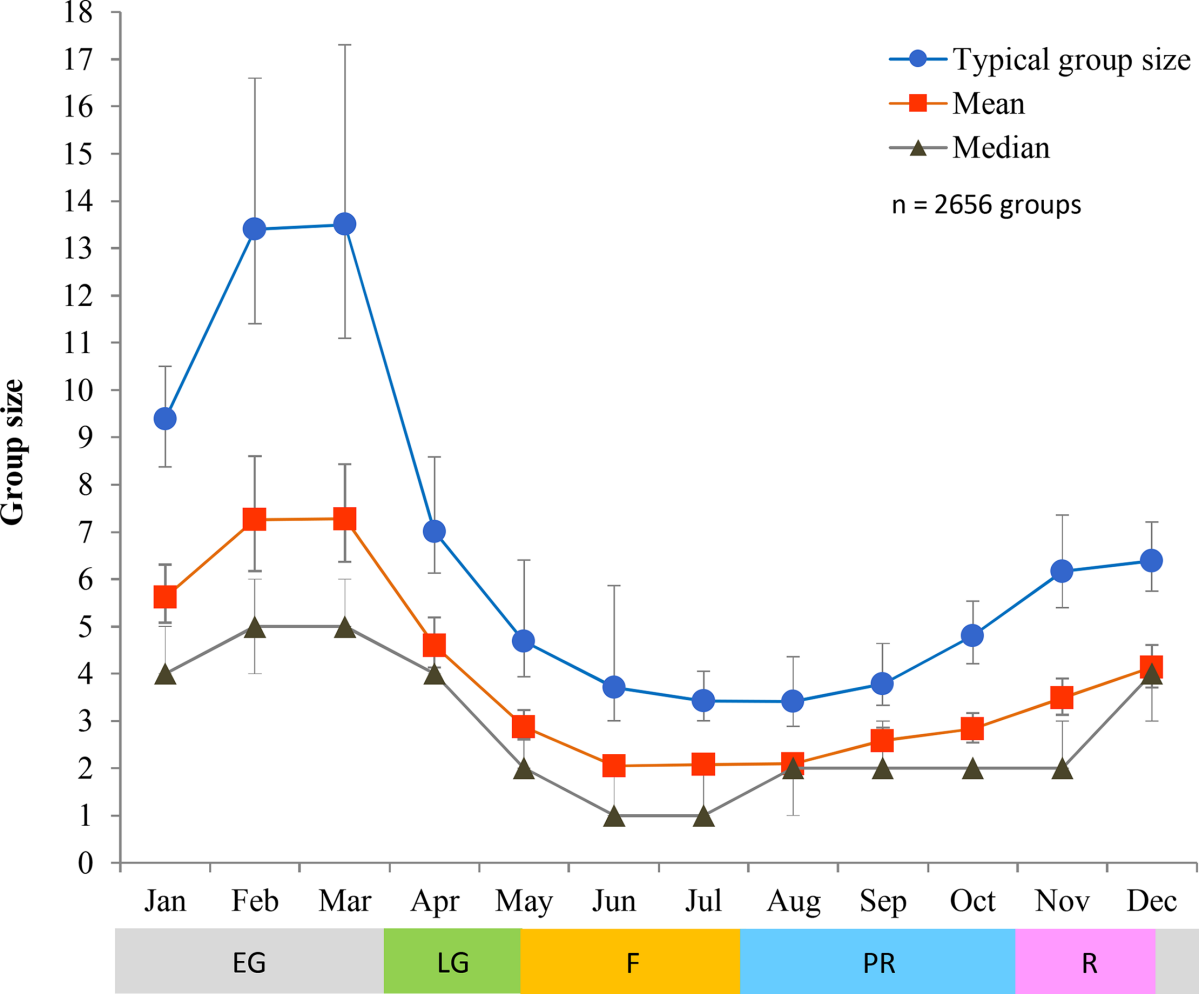
Monthly typical, mean and median group sizes. In typical and mean group sizes bars are 95% confidence intervals (CIs); in median group size CIs range between 95% and 96.2%. See S1 Table for actual values. EG = early gestation, LG = late gestation, F = fawning, PR = pre-rut, and R = rut.

**Fig 3:**
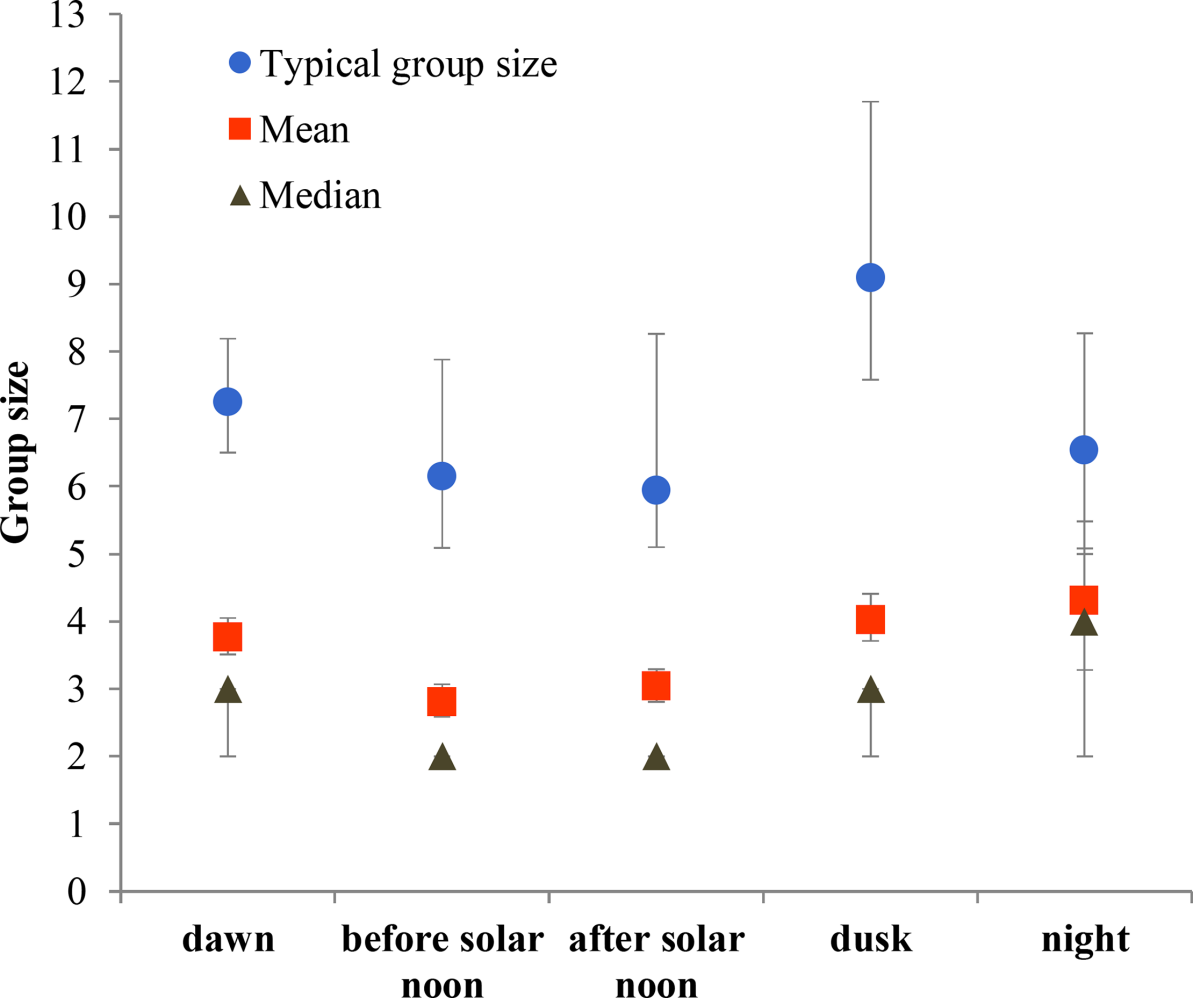
Typical, mean and median group sizes according to time of day. In typical and mean group sizes bars are 95% confidence intervals (CIs); in median group size CIs range between 95% and 96.2%. See S1 Table for detail results.

**Fig 4:**
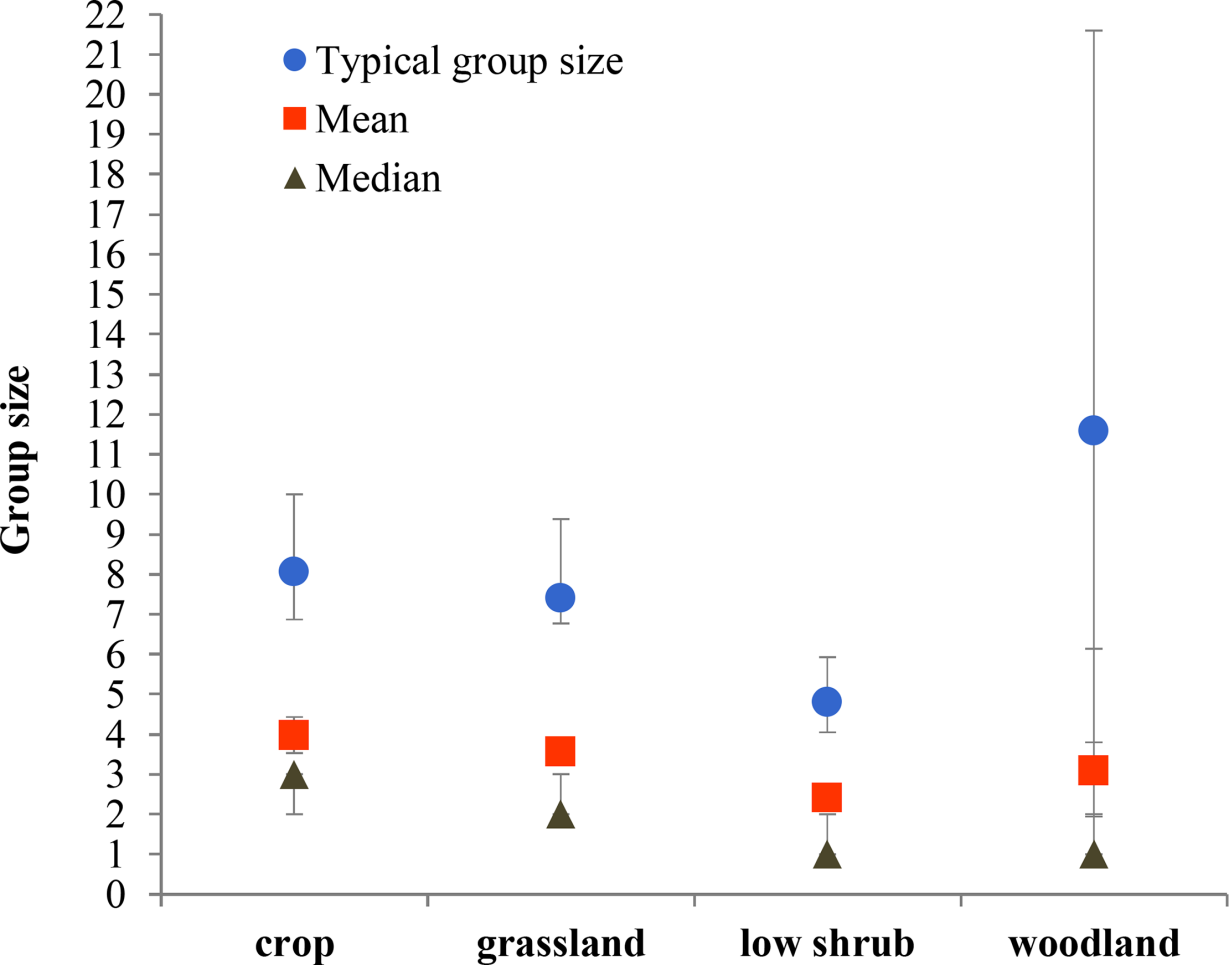
Typical, mean and median group sizes according to habitat. In typical and mean group sizes bars are 95% confidence intervals (CIs); in median group size CIs range between 95% and 96.2%. See S1 Table for detail results.

**Fig 5:**
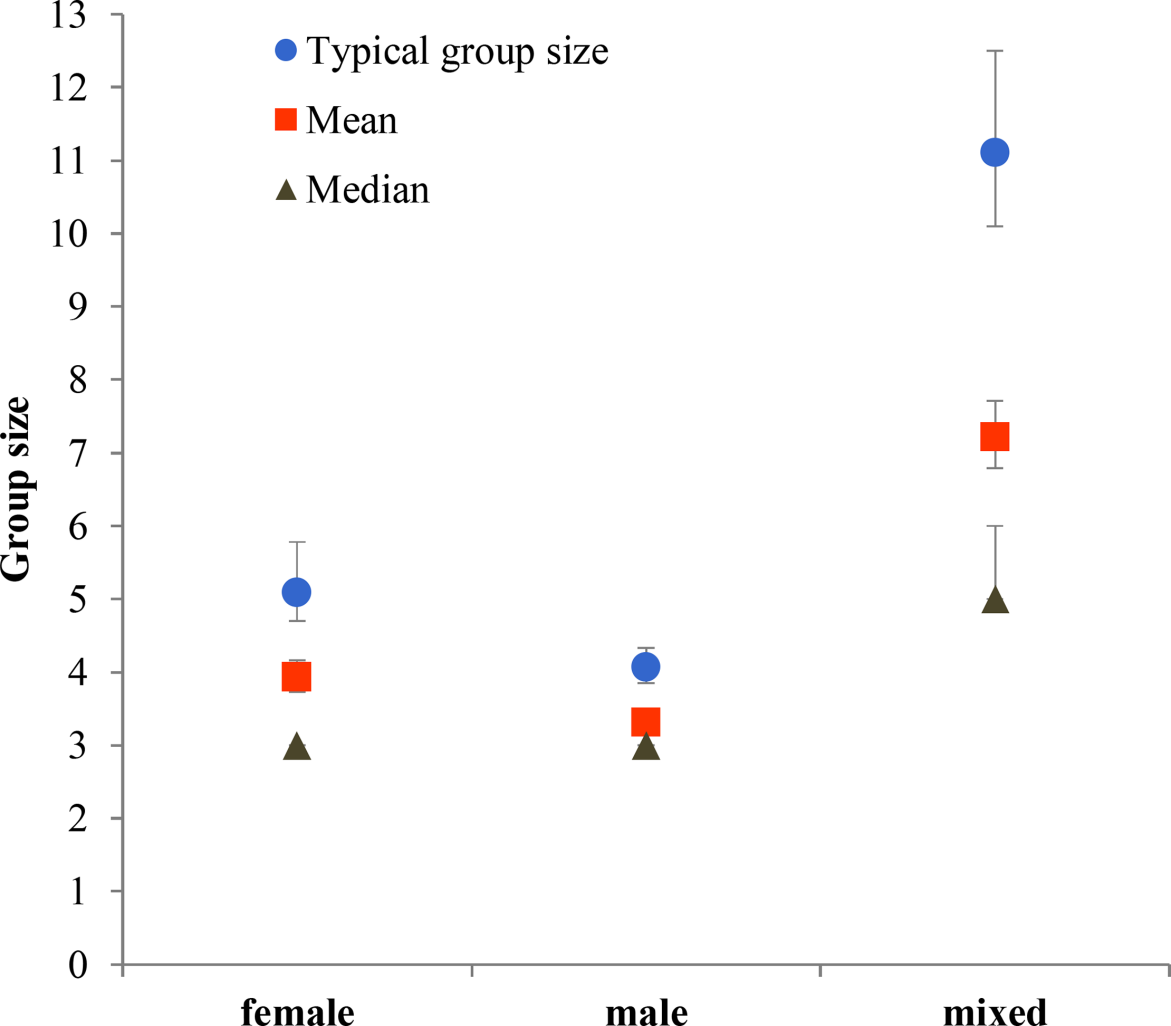
Typical, mean and median group sizes according to group type. In typical and mean group sizes bars are 95% confidence intervals (CIs); in median group size CIs range between 95% and 96.2%. See S1 Table for detail results.

We further described the distribution of group sizes using TGS, mean and median (S1 Table). Results from pairwise comparisons can be found in S2 File. Monthly variation of group size was substantial, and followed a seasonal pattern. Groups were smallest during June and July (fawning) and gradually increased in size until they reached the greatest sizes of the year during February and March (early gestation), then, with the start of late gestation in April, groups rapidly decreased in size (Fig 2). Mean and median group sizes at different times of the day followed a very similar pattern: smallest groups before solar noon and largest groups at dusk (Fig 3); TGS did not vary across different times of day except when before and after solar noon time periods were combined and then dusk had a significantly larger TGS than this combination (S1 Table and S2 File). Groups in low shrub were smaller than those in either crop or grassland (Fig 4), so size increased with habitat openness. Mixed-sex groups were larger than single-sex groups, and female groups were larger than male groups (Fig 5).

## Discussion

CWD, as well as other important infectious diseases in cervids such as tuberculosis and brucellosis, can be transmitted through direct contact between individual animals as well as through the environment. The importance of spatial aggregation of deer, and hence of environmental prions, has been recognized [34], and the relative importance of environmental transmission appears to be stronger than that of direct transmission in theoretical modeling [34, 56]. However, social structure and behavior of the host population influence both transmission pathways, having epidemiological implications in the spread of infections [57]. Therefore, disease models designed to inform CWD control strategies should also include information on social behavioral traits, such as group dynamics of mule deer [26, 27]. In this study we report exhaustive data on mule deer group sizes, and likelihood of group occurrence. In particular, we found that mule deer showing clinical signs of CWD were less likely to be reported in groups than clinically healthy deer after accounting for time of day, habitat, and month of observation.

Based on our study, we cannot identify the mechanisms behind a reduced occurrence of sick individuals in groups. However, there are probable explanations: 1) normal activities daily performed by healthy deer are challenging for sick deer, 2) healthy deer actively avoid grouping with sick deer, and 3) both strategies are occurring. Deer with CWD develop spongiform encephalopathy caused by the accumulation of disease-associated prion proteins [58]. This lesion correlates with clinical signs, such as modification of body postures, reduced awareness, and gradual weight loss with terminal anorexia [5, 8]. As there is no recovery from CWD, these clinical signs affect foraging, mating, and parental care [59, 60], and increase the risk of death due to predation [61, 62] and vehicle collision [63]. One would expect that these signs also affect deer’s ability to enter and remain in a group. Animals are capable of using behavioral immunity as a defense against contagion [20], in other words, healthy individuals can show avoidance of infected animals [21, 64, 65]. For a healthy deer, the cost of increased risk of infection might outweigh the benefits of socializing with sick individuals [17]. Whatever the case, it is clear that presentation of clinical signs of CWD infection is related to mule deer probability of grouping.

It is likely that subtle behavioral changes are apparent to deer and predators, but not to humans, and we do not know at what point in the infectious period deer start showing changes in grouping behavior. Without applying our findings in a dynamic simulation model, it is not possible to fully appreciate the implications of our findings on CWD transmission dynamics. Although CWD models published to date have not included detailed aspects of deer sociality, they recognized the advantages this may have [26, 66]. Remarkably, a study on lobsters infected with PaV1 virus has shown that the only way that empirical observations of virus prevalence over time fit simulations is when behavioral immunity is considered in the model [23]. Adding the information generated by this study on differential grouping likelihoods (i.e. odds of grouping when sick) and group size to current CWD transmission dynamics models should provide new insight on this complex disease.

Similarities of our findings to reports on mule deer in geographical areas free from CWD (California, USA; and Alberta, Canada) include: 1) increase of mean group size with habitat openness [29], 2) comparable overall mean group size (3.5, SD = 2.1, range = 1 to 40, n = 2639 groups) [29], 3) smallest groups in fawning and largest in early gestation [31], and 4) mixed-sex groups larger than groups of females [29, 31], and female groups larger than groups of males [29]. These previous reports were from more natural landscapes: ranchland dominated by prairie and grassland [31] and a state park comprised of upland meadows, tree and chaparral [29]. Our findings extend these observations to agricultural lands comprised of ~50% cropland with grassland and shrub confined to a river valley and associated draws and coulees. Our study is also unique in that it describes groups from both the external observers’ and the group members’ viewpoints using recently developed statistical methodologies [52]. Comparison of results using the three measures of group size did not reveal many differences, which was expected given that mean group size tends to predict mean crowding [49]; however, there were differences in results. For example, any given member of a group would experience a more similar group size across different times of the day than an external observer would. Differences in significance between TGS and mean group size occurred for various months. Moreover, a disease-related variable, such as presence of deer showing clinical signs in a group, was a good predictor of group occurrence, but not of group size. These differences highlight the importance of calculating all three measures of group size, as well as investigating group occurrence, to better describe mule deer sociality.

Two trends are well-documented in cervids: group size increases with habitat openness [67–69], and group size tends to increase with population density [70], but not always [71]. In our study area, mule deer have access to a patch-work of different habitat types, and we demonstrated that group size varies with habitat use. Mule deer might group more in open and flat habitats such as cropland simply because they can detect each other more easily [69, 72], and also because they are more susceptible to predation in non-rugged terrain unless they form groups to dilute the risk of predation. Groups of mule deer are known to merge with other groups and stand their ground as an anti-predator strategy [30].

Female groups were estimated to be larger than male groups in early gestation. One possible explanation is that the proportion of males in mixed-sex groups is greater than that of females, which is not the case in our study (data not shown). A complementary explanation would be that contrary to the way males group, adult females are also joined by their fawns, increasing the group size more rapidly than when males form their groups. Also, female groups are larger in early gestation than in any other season; at that age fawns are with their mother at all times, no longer needing to hide and isolate [31], and are then counted as part of the group when tracking. In ungulate herds, individuals with greater nutritional requirements (e.g. pregnant and lactating females) often lead individuals to whom social cohesion is more crucial and who have larger incentives to avoid group fragmentation (e.g. juveniles and fawns) [31, 73–75]. In terms of disease transmission, as there are repeated reports of greater prevalence of CWD in adult male mule deer [32, 76], group size does not explain this observation. However, larger male home range sizes [77], and increased direct contacts between competing males during pre-rut [78], may be major determinates in CWD transmission. We also wonder if members of groups of females are at a lower risk than members of groups of males, as in the former group type membership can be more stable even though size is larger [31, 79]. Seasonal and habitat use variation in host social behavior, such as large group sizes in winter and in open habitats found in our study, may introduce temporal and spatial patterns in disease transmission. For example, brucellosis seroprevalence in elk is higher at feeding sites in late spring [80] [other examples in 81].

Based on our field observations on mule deer, mother’s hostility towards young males is evident in late gestation. Males then start migrating to new territories [82], forming groups of males that are larger in late gestation than in pre-rut and rut. It is in pre-rut and rut when males show intense rutting activity characterized by tending of females. Typically, single older bigger males find a group of females, monitor their estral status, mate if appropriate, and then move on to the next group of females; young males spend the time alone or with 1 or 2 conspecifics sometimes awaiting for an opportunity to mate [28, 83]. Under these circumstances, size of male groups decreases, and if male group fission rates increase, this may be a time of augmented risk of infection for males as they would become in contact with new individuals.

From our occurrence likelihood, and TGS, mean and median calculations, we found that the largest groups happened in February and March, and the smallest in July. This is not surprising, as similar trends have been previously reported in mule deer [31]. Large winter aggregations are common in social cervids [29, 68], and are perhaps an accentuation of a strategy for protection against predators particularly under conditions that would hinder escape (i.e. deep snow terrain) [84]. Also, in July female mule deer give birth in synchrony, isolating themselves to give birth and hide their offspring [31, 83].

Mule deer are known to move faster (m/min) and to use different habitats at dusk and dawn than at midday [85]. This can enable deer to find conspecifics more easily, and to group as a consequence [69]. In addition, mule deer are known to actively bunch together to defend against coyotes, reducing the risk of predation [30]. Coyote hunts occur more often at dusk, especially in summer and autumn [86], and are more frequently directed to smaller groups [30]. All these might explain why mule deer were not only more likely to be seen in groups, but also in larger groups, at dusk than before solar noon in our study area, where coyotes are their predominant predator.

In summary, we demonstrated that the presentation of clinical signs of disease affect the probability of an individual to be seen in groups, found factors that influence group size, and described mule deer group size distribution. Our data can serve to complement future modeling and parameter estimation; we believe that well-informed spatially- and behaviorally-explicit epidemiological models can serve as important tools to inform and guide CWD management strategies.

## Supporting Information

S1 File Key used to seasonally identify the sex and age classes of mule deer when doing field observations. (DOCX)

S2 File Comparisons of typical, mean and median group sizes among month, season, habitat, time of day, group type, and year. (XLSX)

S1 Table Group size measures of free-ranging mule deer observed from 2008 to 2013 in Antelope Creek, Saskatchewan. (DOCX)
