# Supplementary material for: Infectious Disease and Grouping Patterns in Mule Deer
Source: PLoS One. 2016 Mar 23;11(3):e0150830. doi: 10.1371/journal.pone.0150830 (PMC4805189; doi:10.1371/journal.pone.0150830)
Supplement: S1 File — (DOCX) [file pone.0150830.s001.docx]

**S1 File.** Key used to seasonally identify the sex and age classes of mule deer when doing field observations.

**FAWNING –** From May 16^th^ to July 31^st^ (76 days).

This is the easiest season to recognize differences among ages and sexes.

Males (adults and juveniles) have antlers.

Females are either big (adults) or small (juveniles) with no spotted fur.

Fawns are unmistakable (small and with spotted fur).

| **Adult males (ADM)** | **Adult females (ADF)** |
| --- | --- |
| Have antlers that are:   - Growing and with velvet. - Branched (≥ 2 main tines). - Base thicker than in JVM. - Spread wider than width of ears.   Large body. | Are giving birth 🡪 peak around June 15^th^.  Larger body.  Mature look. |
| **Adults** are ≥2 years and 1 to 2 months old. (24, 25, 26 months; 36, 37, 38 months; and so on). | |
| **Juvenile males (JVM)** | **Juvenile females (JVF)** |
| Have antlers that are:   - Growing and with velvet. - Unbranched (only 2 main tines). - Straight. - Base not as thick as in ADM. - Spread narrower than width of ears.   Slender and long-legged.  Face smaller and narrower than in AD. | Slender and long-legged.  Face smaller and narrower than in AD.  Look quite young, but without white spots and antlers. |
| **Juveniles** are 1 year and 1 to 2 months old. (12, 13, 14 months). | |
| **Fawns – males and females** | |
| Newly born (peak June 15^th^).  Coat with white spots.  Cannot easily tell the difference between sexes. | |

**Fawns** are 1 to 2 months old.

**PRE-RUT –** From August 1^st^ to October 31^st^ (91 days).

It is an easy season to recognize differences among ages and sexes.

Males have antlers all the time.

Be careful with ADF and JVF, they start to look similar.

Fawns in August are still unmistakable (spotted), but by September they lose their spots.

| **Adult males (ADM)** | **Adult females (ADF)** |
| --- | --- |
| Have antlers that are:   - Fully grown. - Will lose the velvet through the season (no velvet by Oct). - Branched (≥ 2 main tines). - Base thicker than in JVM. - Spread wider than width of ears.   Large body. | Are nursing.  Larger body.  Do not look young at all.  Most probably seen with fawns. |
| **Adults** are ≥2 years and 2 to 5 months old. (26, 27, 28, 29 months; 38, 39, 40, 41 months; and so on). | |
| **JVM** | **JVF** |
| Have antlers that are:   - Fully grown. - Will lose the velvet through the season (no velvet by Oct). - Unbranched (only 2 main tines). - Straight. - Base not as thick as in ADM. - Spread narrower than width of ears.   Slender and long-legged.  Face smaller and narrower than in AD. | Slender and long-legged.  Face smaller and narrower than in AD. |
| **Juveniles** are 1 year and 2 to 5 months old. (14, 15, 16, 17 months). | |
| **Fawns – males and females** | |
| Spotted fur only in August. They lose their spots by September.  In October they get a winter coat that makes them look fluffy and with round faces.  Sometimes the antler buds can be seen in males.  **Fawns** are between 2 and 5 months old. | |

**RUT-** From November 1^st^ to December 15^th^ (44 days).

It is an easy season to recognize differences among sexes.

Males have antlers all the time.

Be careful with ADF and JVF, they look similar.

Fawns are easy to identify depending on the angle of the picture.

| **Adult males (ADM)** | **Adult females (ADF)** |
| --- | --- |
| Massive neck swelling (more pronounced than in JVM).  Maybe tarsal glands more stained than in JVM.  Have antlers that are:   - Grown with no velvet. - Branched (≥ 2 main tines). - Base diameter larger than in JVM. - Spread wider than width of ears.   Large body. | Still nursing.  Larger body.  Most probably seen with fawns and followed by males. |
| **Adults** are ≥2 years and 5 to 6.5 months old. (29, 30 months; 41, 42 months; and so on). | |
| **Juvenile males (JVM)** | **Juvenile females (JVF)** |
| Neck swelling less pronounced than in ADM.  May be tarsal glands with lighter staining than in ADM.  Have antlers that are:   - Grown with no velvet. - Unbranched (only 2 main tines). - Straight. - Base not as large as in ADM. - Spread narrower than width of ears.   Their bodies are less slender than in pre-rut, but still more slender than ADM. | Slender and long-legged, but quite similar to ADF.  Face smaller and narrower than in AD.  They will also be followed by males. |
| **Juveniles** are 1 year and 5 to 6.5 months old. (17, 18 months). | |
| **Fawns – males and females** | |
| Winter coat that makes them look fluffy and with round faces.  Sometimes the antler buds can be seen in males.  **Fawns** are between 5 and 6.5 months old. | |

**EARLY GESTATION –** From December 16^th^ to March 31^st^ (105 days).

Difficulties in this season:

Adult and juvenile males will lose their antlers, so they are very similar to females.

Adult and juvenile females are very similar.

Recommendation: compare deer from different pictures before making a final decision.

| **Adult males (ADM)** | **Adult females (ADF)** |
| --- | --- |
| Most of them will lose their antlers by the end of February and beginning of March, thus they may have blood stains on frontal area of the head.  If with antlers: same as in rut.  Neck swelling decreases, but neck is still wider than in JVM. | Still nursing.  Larger body than JV. |
| **Adults** are ≥2 years and 6.5 to 10 months old. (30, 31, 32, 33, 34 months; 42, 43, 44, 45, 46 months; and so on). | |
| **Juvenile males (JVM)** | **Juvenile females (JVF)** |
| Most of them will lose their antlers by the end of February and beginning of March, thus they may have blood stains on frontal area of the head.  If with antlers: same as in rut.  Neck is not as wide as in ADM. | Slender and long-legged, but quite similar to ADF.  Face smaller and narrower than in AD. |
| **Juveniles** are 1 year and 6.5 to 10 months old. (18, 19, 20, 21, 22 months). | |
| **Fawns – males and females** | |
| Winter coat makes them look fluffy and with round faces.  Sometimes the antler buds can be seen in males.  **Fawns** are 6.5 to 10 months old. | |

**LATE GESTATION –** From April 1^st^ to May 15^th^ (44 days).

This is the most difficult season to recognize differences among ages and sexes (except for fawns).

All age classes start looking similar to the next age class: fawns look quite juvenile (still with short front), juveniles are big (like small adults), adult males are slim (no big neck), and adult females look bigger than juveniles.

Antler buds in fawns look larger (in length mainly), than in juveniles and adults.

NOTE: Important is to notice that all fawns needed to be classified as juveniles, and all juveniles as adults when registering data for group sighting purposes. This is the only season in which this alteration was made.

| **Adult males (ADM)** | **Adult females (ADF)** |
| --- | --- |
| No antlers.  Neck is not swollen.  No signs of antler shedding (no blood). | Large long faces.  They look bigger than juveniles. |
| **Adults** are ≥2 years and 10 to 11.5 months old. (34, 35 months; 46, 47 months; and so on). | |
| **Juvenile males (JVM)** | **Juvenile females (JVF)** |
| No antlers.  Just about to became adults, but smaller. | Small adults. |
| **Juveniles** are just about to become adults; they are almost 2 years old (1 year and 10 to 11.5 months old). | |
| **Male fawns** | **Female fawns** |
| Their antlers are growing and even when they are very small, they may look bigger than those in AD and JV males.  Face is rounder than in JV. | Any deer that has a fawn face and has no antlers is a female fawn. |
| **Fawns** are almost 1 year old (between 10 and 11.5 months old). | |

**References** This document is based on field observations done on Rocky Mountain mule deer (*Odocoileus hemiounus hemiounus*) in southern Saskatchewan, Canada, from 2009 to 2012 and is adapted from descriptions made by Raymond F. Dasmann (1956) and Valerius Geist (1998).

Dasmann RF, Taber RD (1956) Determining structure in Columbian black-tailed deer populations. J Wildlife Manage 20:78-83.

Geist V (1998) White-tailed deer and mule deer. In: Geist V (ed) Deer of the world: Their evolution, behaviour, and ecology. 1st edn. Stackpole Books, United States of America, pp 255-301
