# Supplementary material for: Infectious Disease and Grouping Patterns in Mule Deer
Source: PLoS One. 2016 Mar 23;11(3):e0150830. doi: 10.1371/journal.pone.0150830 (PMC4805189; doi:10.1371/journal.pone.0150830)
Supplement: S1 Table — (DOCX) [file pone.0150830.s003.docx]

**S2 Table.** Group size measures of free-ranging mule deer observed from 2007 to 2013 in Antelope Creek, Saskatchewan.

| Variable | Number of individuals | Number of groups | TGS | TGS lower CI^a^ | TGS upper CI^a^ | Mean | Mean lower CI^b^ | Mean upper CI^b^ | SD^c^ | Median | Median lower CI | Median upper CI | Actual %CI^d^ |
| --- | --- | --- | --- | --- | --- | --- | --- | --- | --- | --- | --- | --- | --- |
| All groups | 9177 | 2656 | 7.3 | 6.8 | 8.1 | 3.5 |  |  | 3.7 | 2 | 2 | 2 | 95.0 |
| **Time of day** |  |  |  |  |  |  |  |  |  |  |  |  |  |
| Dawn | 3732 | 727 | 7.3 | 6.5 | 8.2 | 3.8 | 3.5 | 4.1 | 3.6 | 3 | 2 | 3 | 95.6 |
| Before solar noon | 1781 | 636 | 6.2 | 5.1 | 7.9 | 2.8 | 2.6 | 3.1 | 3.1 | 2 | 2 | 2 | 95.4 |
| After solar noon | 1846 | 607 | 5.9 | 5.1 | 8.3 | 3.0 | 2.8 | 3.3 | 3.0 | 2 | 2 | 2 | 95.6 |
| Dusk | 2541 | 632 | 9.1 | 7.6 | 11.7 | 4.0 | 3.7 | 4.4 | 4.5 | 3 | 2 | 3 | 95.6 |
| Night | 125 | 29 | 6.5 | 5.1 | 8.3 | 4.3 | 3.3 | 5.5 | 3.2 | 4 | 2 | 5 | 96.5 |
| Day^e^ | 3627 | 1243 | 6.0 | 5.4 | 7.1 | 2.9 | 2.8 | 3.1 | 3.0 | 2 | 2 | 2 | 95.0 |
| **Habitat** |  |  |  |  |  |  |  |  |  |  |  |  |  |
| Cropland | 1116 | 282 | 8.1 | 6.9 | 10.0 | 4.0 | 3.5 | 4.4 | 4.0 | 3 | 2 | 3 | 95.8 |
| Grassland | 6800 | 1907 | 7.4 | 6.8 | 9.4 | 3.6 | 3.4 | 3.7 | 3.7 | 2 | 2 | 3 | 95.0 |
| Woodland | 102 | 33 | 11.6 | 3.8 | 21.6 | 3.1 | 1.9 | 6.1 | 5.2 | 1 | 1 | 2 | 96.0 |
| Low shrub | 904 | 373 | 4.8 | 4.1 | 5.9 | 2.4 | 2.2 | 2.7 | 2.4 | 1 | 1 | 2 | 95.1 |
| Other | 255 | 61 | 9.7 | 6.3 | 14.7 | 4.2 | 3.3 | 5.9 | 4.8 | 2 | 2 | 4 | 96.3 |
| **Year** |  |  |  |  |  |  |  |  |  |  |  |  |  |
| 2009 | 1743 | 521 | 6.2 | 5.5 | 7.4 | 3.4 | 3.1 | 3.6 | 3.1 | 2 | 2 | 3 | 95.2 |
| 2010 | 3204 | 995 | 5.8 | 5.4 | 6.4 | 3.2 | 3.1 | 3.4 | 2.9 | 2 | 2 | 2 | 95.0 |
| 2011 | 3405 | 884 | 9.7 | 8.5 | 11.5 | 3.9 | 3.6 | 4.2 | 4.8 | 2 | 2 | 2 | 95.0 |
| 2012 | 825 | 256 | 5.8 | 5.1 | 6.7 | 3.2 | 2.9 | 3.6 | 2.9 | 2 | 2 | 3 | 95.4 |
| **Season** |  |  |  |  |  |  |  |  |  |  |  |  |  |
| Early gestation | 3367 | 534 | 11.7 | 10.5 | 13.3 | 6.3 | 5.9 | 6.8 | 5.8 | 5 | 4 | 5 | 95.4 |
| Late gestation | 1019 | 242 | 6.7 | 6.0 | 8.0 | 4.2 | 3.8 | 4.7 | 3.3 | 4 | 3 | 4 | 95.9 |
| Fawning | 1797 | 844 | 3.5 | 3.2 | 4.6 | 2.1 | 2.0 | 2.3 | 1.7 | 2 | 1 | 2 | 95.4 |
| Pre-rut | 1710 | 693 | 4.0 | 3.7 | 4.4 | 2.5 | 2.3 | 2.6 | 2.0 | 2 | 2 | 2 | 95.3 |
| Rut | 1284 | 343 | 6.2 | 5.6 | 6.9 | 3.7 | 3.5 | 4.1 | 3.0 | 3 | 2 | 3 | 95.8 |
|  |  |  |  |  |  |  |  |  |  |  |  |  |  |
| **Month** |  |  |  |  |  |  |  |  |  |  |  |  |  |
| January | 1096 | 195 | 9.4 | 8.4 | 10.5 | 5.6 | 5.1 | 6.3 | 4.6 | 4 | 4 | 5 | 95.8 |
| February | 908 | 125 | 13.4 | 11.4 | 16.6 | 7.3 | 6.2 | 8.6 | 6.7 | 5 | 4 | 6 | 95.1 |
| March | 1135 | 156 | 13.5 | 11.1 | 17.3 | 7.3 | 6.4 | 8.4 | 6.7 | 5 | 5 | 6 | 95.7 |
| April | 726 | 158 | 7.0 | 6.1 | 8.6 | 4.6 | 4.1 | 5.2 | 3.3 | 4 | 4 | 4 | 95.1 |
| May | 605 | 210 | 4.7 | 3.9 | 6.4 | 2.9 | 2.6 | 3.2 | 2.3 | 2 | 2 | 3 | 95.8 |
| June | 795 | 387 | 3.7 | 3.0 | 5.9 | 2.1 | 1.9 | 2.3 | 1.8 | 1 | 1 | 2 | 95.8 |
| July | 690 | 331 | 3.4 | 3.0 | 4.1 | 2.1 | 1.9 | 2.3 | 1.7 | 1 | 1 | 2 | 95.0 |
| August | 575 | 275 | 3.4 | 2.9 | 4.4 | 2.1 | 1.9 | 2.3 | 1.7 | 2 | 1 | 2 | 95.4 |
| September | 516 | 200 | 3.8 | 3.3 | 4.6 | 2.6 | 2.4 | 2.9 | 1.8 | 2 | 2 | 3 | 96.2 |
| October | 619 | 218 | 4.8 | 4.2 | 5.5 | 2.8 | 2.5 | 3.2 | 2.4 | 2 | 2 | 2 | 95.5 |
| November | 768 | 221 | 6.2 | 5.4 | 7.4 | 3.5 | 3.1 | 3.9 | 3.1 | 2 | 2 | 3 | 96.0 |
| December | 744 | 180 | 6.4 | 5.8 | 7.2 | 4.1 | 3.7 | 4.6 | 3.1 | 4 | 3 | 4 | 95.7 |
| **Group type** |  |  |  |  |  |  |  |  |  |  |  |  |  |
| Adult female-fawn/JV dyad | 316 | 158 | 2.0 | NA | NA | 2.0 | NA | NA | NA | 2 | NA | NA | NA |
| Solitary male group | 510 | 510 | 1.0 | NA | NA | 1.0 | NA | NA | NA | 1 | NA | NA | NA |
| Solitary female group | 403 | 403 | 1.0 | NA | NA | 1.0 | NA | NA | NA | 1 | NA | NA | NA |
| Group of males | 1706 | 515 | 4.1 | 3.9 | 4.3 | 3.3 | 3.2 | 3.5 | 1.6 | 3 | 3 | 3 | 95.3 |
| Group of females | 1604 | 408 | 5.1 | 4.7 | 5.8 | 3.9 | 3.7 | 4.2 | 2.1 | 3 | 3 | 3 | 95.8 |
| Mixed-sex group | 3852 | 534 | 11.1 | 10.1 | 12.5 | 7.2 | 6.8 | 7.7 | 5.3 | 5 | 5 | 6 | 95.4 |
| Group with unknowns | 786 | 128 | 10.2 | 8.5 | 12.4 | 6.1 | 5.4 | 7.0 | 5.0 | 4 | 4 | 6 | 95.1 |
|  |  |  |  |  |  |  |  |  |  |  |  |  |  |

Abbreviations: TGS is typical group size; CI is confidence interval; SD is standard deviation; BCa is biased-corrected and accelerated bootstrap; JV is juvenile.

^a^ 95% CI for the TGS calculated by the BCa method with 5000 bootstrap replications. ^b^ 95% CI for the mean calculated by the Bca method with 2000 bootstrap replications.

^c^ SD obtained from the bootstrap 2-sample *t*-test for comparison of means. ^d^ When it was not possible to construct exactly 90, 95 or 99% CI, the shortest interval with at least the desired confidence level was selected, and the exact (actual) level is reported. ^e^ Day was a combination of before and after solar noon periods.
